# Clinical and whole-exome sequencing findings in two siblings from Hani ethnic minority with congenital glycosylation disorders

**DOI:** 10.1186/s12881-019-0902-z

**Published:** 2019-11-14

**Authors:** Zhen Zhang, Ti-Long Huang, Jing Ma, Wen-Ji He, Huaiyu Gu

**Affiliations:** 1 Department of Human Anatomy, Zhongshan School of Medicine, Sun Yat-Sen University, Guangzhou, 510080 People’s Republic of China; 2 Yunnan Key Laboratory of Childrenʼs Major Disease Research, and Yunnan Institute of Pediatrics, Kunming Childrenʼs Hospital, Kunming, 650228 Yunnan China; 3 Department of Hematology, Kunming Children’s Hospital, Kunming Medical University, Kunming, Yunnan 650228 People’s Republic of China; 4 Department of Otolaryngology-Head and Neck Surgery, Kunming Children’s Hospital, Kunming Medical University, Kunming, Yunnan 650228 People’s Republic of China

**Keywords:** Novel variants, PMM2-CDG, Children, Hani ethnic minority

## Abstract

**Background:**

PMM2-CDG, is the most common N-linked glycosylation disorder and subtype among all CDG syndromes, which are a series of genetic disorders involving the synthesis and attachment of glycoproteins and glycolipid glycans. The mutations of PMM2-CDG might lead to the loss of *PMM2*, which is responsible for the conversion of mannose 6- phosphate into mannose 1-phosphate. Most patients with PMM2-CDG have central nervous system involvement, abnormal coagulation, and hepatopathy. The neurological symptoms of PMM2-CDG are intellectual disability (ID), cerebellar ataxia, and peripheral neuropathy. Now, over 100 new CDG cases have been reported. However, each type of CDG is very rare, and CDGs are problematic to diagnose. In addition, few CDGs have been reported in the Chinese population.

**Case presentation:**

Here we present a Hani ethnic minority family including two siblings with congenital glycosylation disorders. Whole-exome sequencing revealed compound heterozygous for one novel mutation (c.241–242 del variant) and previously reported mutation (c.395 T > C) in gene of *PMM2.* Two mutations were found in proband and her sibling by whole-exome sequencing. The mutations were identified in this family by Sanger sequencing and no mutations were detected in the normal control.

**Conclusions:**

This is the first report to describe mutations in two siblings of Hani ethnic minority which is one of five ethnic groups found only in Yunnan with a population of more than 1 million.

## Background

CDGs (Congenital disorder of glycosylation), which are clinically characterized by the defective glycosylation of proteins and lipids, often cause serious, sometimes fatal, malfunctions of several different organ systems, especially the nervous, muscular, immune, hepatic and gastrointestinal systems [1, 2]. In 1991, these diseases were originally called carbohydrate-deficient glycoprotein syndromes and encompassed all types of disorders involving glycoconjugates [3]. In 1999, CDGSs were renamed CDGs [4]. By 2013, the number of CDGs had increased dramatically, with a new glycosylation disorder reported every 17 days, and since then, the reported number of CDGs has surpassed 100, with on-going characterization of new subsets [5, 6]. CDGs, which are autosomal recessive inherited disorders, affect glycan synthesis and include disorders involving protein N-glycosylation, protein O-glycosylation, lipid and glycosylphosphatidylinositol anchor glycosylation and multiple glycosylation pathways [7, 8]. There are two subgroups of protein N-glycosylation diseases, type I CDG (CDG-I) and type II CDG (CDG-II). The largest group, CDG-I, results from defects either in the assembly of the lipid-linked oligosaccharide precursor Glc3Man9GlcNAc2-P-P-dolichol or in its transfer to the nascent polypeptides in the endoplasmic reticulum [9]. The second subgroup, CDG-II, results from the remodelling of protein-bound glycan chains or from alterations in their processing [10].

The most common N-linked glycosylation disorder, PMM2-CDG, which is recessively inherited and is also one of the most common subtypes of CDGs overall, is caused by the lack of phosphomannomutase 2 (*PMM2*). Mutations in *PMM2* might reduce the activity of the phosphomannomutase enzyme, which converts mannose 6-phosphate into mannose 1-phosphate [11, 12]. Depending on the affected organs, there is a wide variety of clinical manifestations that characterize PMM2-CDG, and the PMM2-CDG phenotype varies from very severe to mild [13]. The symptoms of PPM2-CDG include intellectual disability, cerebellar dysfunction, and hypotonia [12]. However, the clinical signs indicating each subtype of PMM2-CDG are difficult to discern because clinical variability is seen not only among patients with the same *PMM2* genotypes but also between affected siblings and monozygotic twins [14]. Additionally, few CDGs have been reported in the Chinese population, especially among children in Yunnan Province. Congenital disorders of glycosylation with normal cognitive development in children from the Yun-Gui Plateau have not been systemically studied, and their pathophysiology is not fully understood.

Here, we identified and reported the mutations in one Hani ethnic minority family with two siblings affected from Yunnan, which has been inhabited by 26 different ethnicities throughout history.

## Case presentation

### Patient recruitment

One non-consanguineous Honghe Hani ethnic minority family from the Yun-Gui Plateau was recruited by the Children’s Hospital of Kunming Medical University for genetic diagnosis. In this family, two siblings suffered from congenital disorders of glycosylation, but the parents and other members were normal. Additionally, 30 individuals of normal control without associated hereditary diseases were enrolled in this study, including 20 males and10 females. Blood samples were collected on February 7th, 2017.This study was approved by the Ethics Committee of the Children’s Hospital of Kunming Medical University, and written informed consent was obtained from the participants or their guardians.

#### Clinical presentation

Pedigree of this family was told by the parents of proband. This family includes two affected siblings with CDGs, an 8-year-old girl (the proband) and a 2-year-old boy. The other members of this family are normal.

**Fig. 1:**
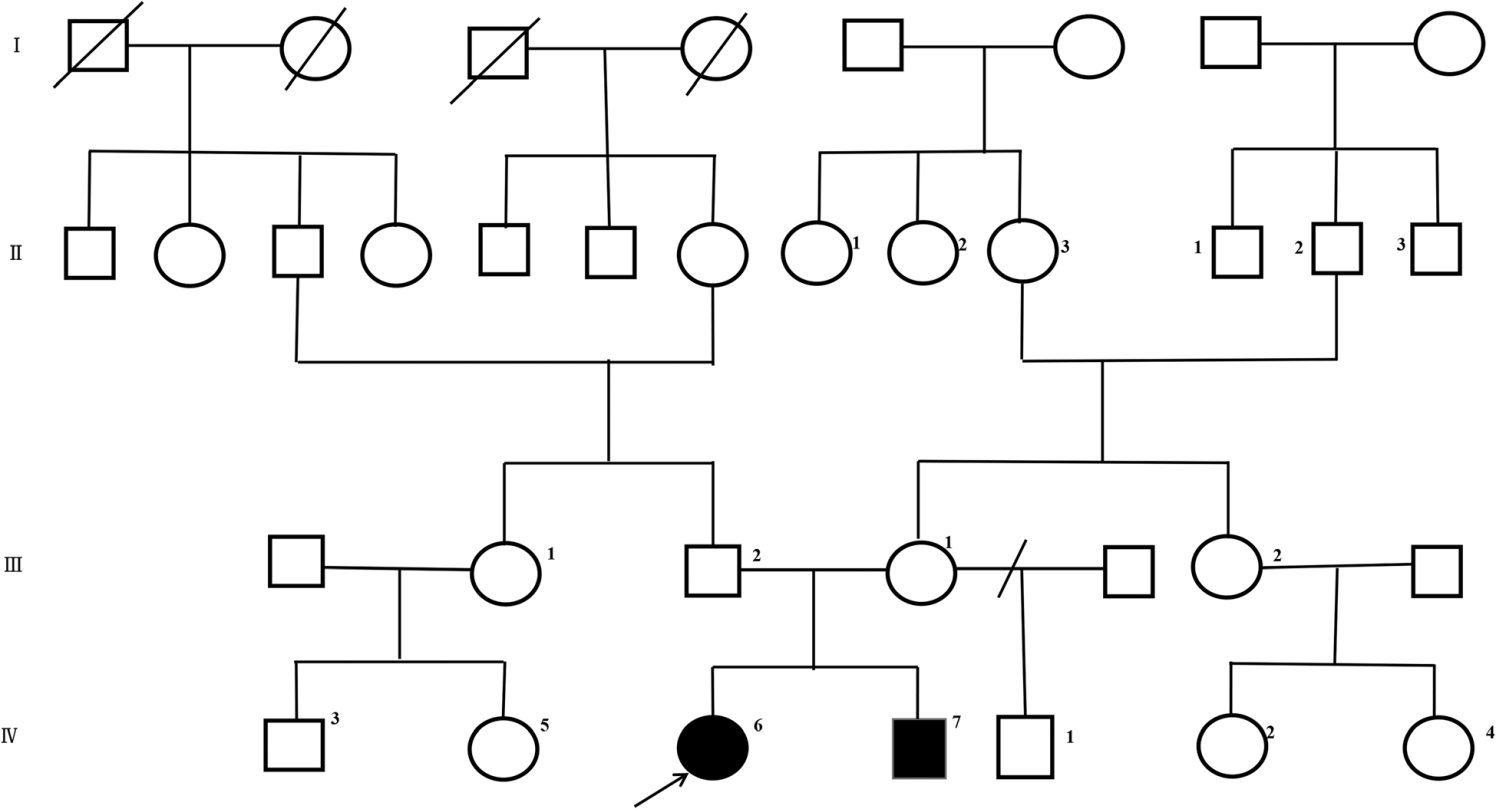
Pedigree of family 1 with CDG. Unaffected subjects are denoted as blank while affected subjects are represented with darkened symbols. The arrow indicates the proband

The proband (IV6 in Fig. 1), was born at 40 weeks of gestation without asphyxia by caesarean delivery to healthy Hani Chinese parents. The patient’s birthweight was 2.9 kg, and she was first diagnosed with an inherited metabolic disease at the age of 8 months. When she was 91 months old, she presented with delayed motor skills, muscular hypotonia, strabismus, an underdeveloped cerebellum. She also had blood clotting disorders, intellectual disability and a failure to gain weight or thrive. She was unable to walk independently. Her speech development was delayed and had dysarthria.

Her younger affected sibling, a 2-year-old boy (IV7 in Fig. 1), had delayed motor skills, muscular hypotonia, strabismus, an underdeveloped cerebellum. He also had intellectual disability and a failure to gain weight or thrive. He was unable to walk independently. His speech development was delayed and had dysarthria.

**Fig. 2:**
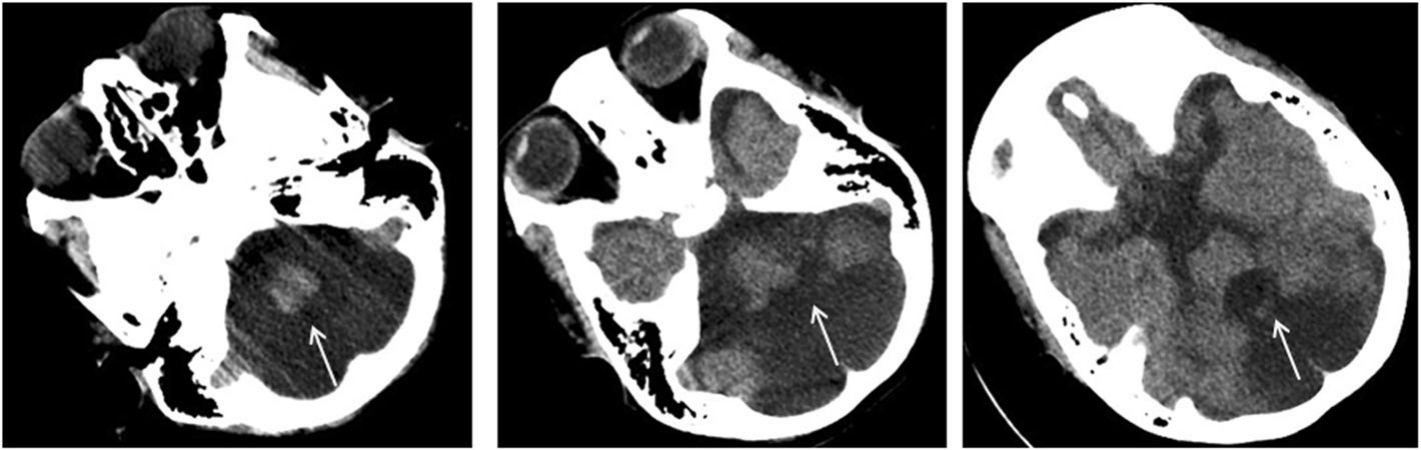
CT imaging showed the unclear of cerebellar vermis and enlarged fourth ventricle of the proband

The parents were not a consanguineous couple, and they denied any family history of CDGs. The clinical characteristics of this family are summarized in Table 1 and the CT of the proband is shown in Fig. 2.

**Table 1:** Summary of the clinical presentation of patients with mutations

	The proband	The younger sibling
Age at last reported assessment	91 Months	19 Months
Birth weight (Kg)	2.9	NA
Delayed motor skills	+	+
Muscular hypotonia	+	+
Strabismus	+	+
Underdeveloped cerebellum	+	NA
Blood clotting disorders	+	–
Intellectual disability	+	–
Failure to gain weight or thrive	+	+
Feeding difficulties	–	–
Speech delay / absence	+	+
Febrile seizures	+	–
Seizures / epilepsy	–	–
Dysmorphic facies	–	–
Abnormalities of the hands or feet	Normal	Normal
Abnormalities of the spine or chest	Normal	Normal
Gastrointestinal symptoms	Normal	Normal
Cardiac	Normal	Normal
Blood platelets	21 × 10^9^/L	NA
CT	Unnormal	**NA**

*NA* Not available

### Whole-exome sequencing (WES)

Using Bcl2Fastq software (Bcl2Fastq 2.18.0.12, Illumina, Inc.), raw image files were processed for base calling and raw data generation. Then, Short Oligonucleotide Analysis Package (SOAP) aligner software (SOAP2.21,soap.genomics.org.cn/soapsnp.html) was used to align the clean reads to the reference human genome (UCSC hg19, http://genome.ucsc.edu/). Polymerase chain reaction (PCR) duplicates were removed by the Picard programme [15, 16]. The single nucleotide polymorphisms (SNPs) were determined by the SOAPsnp programme [17]. The reads were realigned by Burrows-Wheeler Aligner (BWA) software 0.7.15, and the deletions and insertions (indels) were detected by Genome Analysis Toolkit software 3.7. In addition, the identified indel SNPs were annotated using the Exome-assistant programme (http://122.228.158.106/exomeassistant). To determine their pathogenicity, non-synonymous variants were evaluated by four algorithms, namely, PolyPhen (http://genetics.bwh.harvard.edu/pph2/), Protein Analysis Through Evolutionary Relationships (PANTHER, www.pantherdb.org), Sorting Intolerant from Tolerant [SIFT, (http://sift.jcvi.org/)] and Pathogenic Mutation Prediction (Pmut; http://mmb.pcb.ub.es/PMut/).

WES was used to sequence the genes of the these two siblings. Exome sequencing produced about 160.55 and 80.6 million reads with a read length of 143 and 149 bp in the proband and proband’s sibling, respectively. There were 160.05 and 80.52 million reads aligned to the human genome respectively; 22,781.9 and 12,001.58 Mb were mapped to the target region with a mean coverage of 99.68 and 99.87 respectively. 33,532 and 42,423 SNPs, including 12,392 and 12,379 non-synonymous SNPs in the coding sequence and 1033 and 1017in the splice sites, were respectively detected. 1443 and 1466 indels, including 597and 587 in the coding sequence and 322and 358 in the splice sites, were respectively identified.

The compound heterozygous for PMM2 c.241–-242 del (in exon 3) and c.395 T > C (in exon 5) mutations were found in these siblings (Table 2).

**Table 2:** Identified mutations

Gene	Exon	Nucleotide mutations	Allele state	Protein effect	dbSNP ID	Mutation type	Phenotype
*PMM2*	3	c.241-242del	het	p.L82Vfs*2	novel	frameshift	congenital disorder of glycosylation
*PMM2*	5	c.395 T > C	het	p.I132T	known	nonsynonymous	

#### Identification of pathogenic mutations

**Fig. 3:**
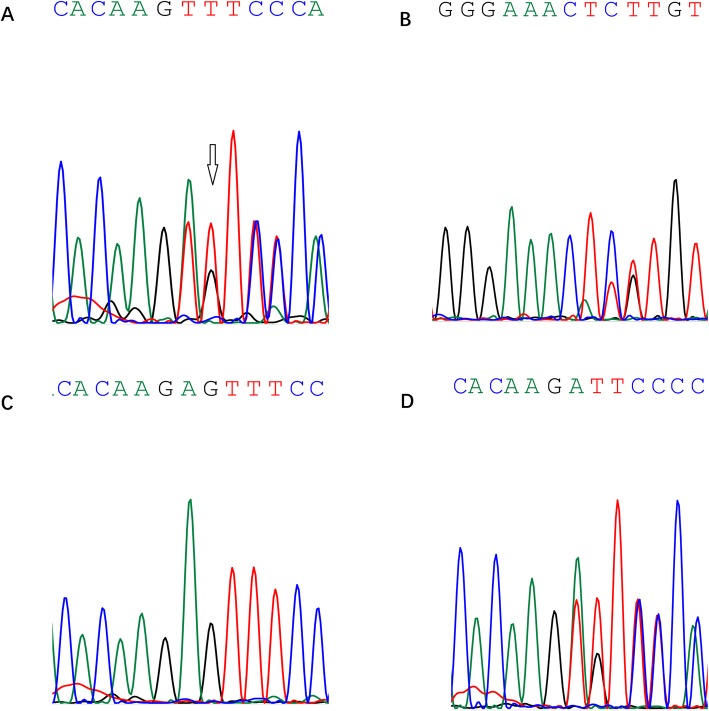
Partial electropherograms of the genomic region covering the genes: The heterozygous *PMM2* c.241-242del variant. Arrows denote the mutations. **a** the proband; **b** the younger affected brother; **c** the unaffected father; **d** the unaffected mother

The heterozygous *PMM2* c.241–242 del and c.395 T > C mutations identified in this family were confirmed by Sanger sequencing: The heterozygous *PMM2 c.241–242* del and c.395 T > C were identified in the proband’s affected brother (Fig. 3); The heterozygous *PMM2 c.241–242* del and c.395 T > C were identified in the proband’s unaffected mother and unaffected father, respectively. Additionally, these two variants were absent in 30 normal control individuals.

## Discussion and conclusions

In humans, *PMM1* and *PMM2* are two paralogous enzymes [18]. *PMM1* has never been associated with human disease, while mutations in *PMM2* cause PMM2-CDG. In humans, homozygous mutations of *PMM2* have never been observed. This indicates that the total absence of the *PMM2* enzyme is not compatible with life [19]. These findings have been confirmed in mice, and the disruption of *PMM1* results in no apparent deleterious effects in mouse embryos [20], whereas the disruption of *PMM2* causes early embryonic lethality [21].

PMM2-CDG, a disease with defective N-glycan assembly, is caused by the lack of *PMM2* activity [22]. The mutation of the *PMM2* gene results in enzymatic deficiency, reducing the amount of GDP-mannose, which is required for the synthesis of the lipid-linked oligosaccharide precursor. The common symptoms are intellectual disability, cerebellar dysfunction, and hypotonia [23]. More than 50% of affected individuals have intellectual disabilities with scores ranging from very low to below average [12, 24].

In our study, when the proband saw the doctor to cure refractory thrombocytopenia, the common.

symptoms of CDG, such as intellectual disability, hypotonia, and cerebellar dysfunction, were observed in the two affected siblings via inquiring medical history. Though these siblings suffered from this disease, their parents didn’t know which affected their children’s intellectual disabilities and agreed to accepted gene testing. So, WES was used to screen genes of these siblings. The heterozygous *PMM2* c.241–242 del variant and c.395 T > C were observed in these siblings. Then, using Sanger sequencing, the heterozygous *PMM2* c.241–242 del variant and c.395 T > C were identified in unaffected mother and unaffected father, respectively.

In this family, the proband (IV6) and her younger sibling (IV7) were affected while their elder half-brother is normal (see Fig. 1 IV1). To our best knowledge, the compound heterozygous for one mutation (c.241–242 del variant) and mutation (c.395 T > C) in gene of *PMM2* might account for these two sibling’s CDGs.

According to PubMed, there are over 100 CDGs [25], but reports of CDGs are uncommon in Asian patients [26]; there have been few reports of CDGs in Chinese patients, although one paper reported two Chinese female infants with CDGs who had PMM2 gene mutations [27]. This might account that doctor didn’t give parent “a definitive diagnosis” when the proband was the age of 8 months.

To the best of our knowledge, this is the first report of individuals with CDGs in Yunnan Province which has been inhabited by 26 ethnic minority groups throughout history.

Of these 26 ethnic minority groups, 15 are found only in Yunnan and five ethnic groups have a population of more than 1 million. Hani ethnic minority group is one of five and more than 95% of their population live in Yunnan. In our study, this Hani ethnic minority family has two children with PMM2-CDG; the proband did not receive a molecular diagnosis, so this family did not obtain a prenatal genetic diagnosis before another child was born. This might be due to the fact that the counties and towns in Yunnan Province are insular, resulting in low awareness of this disorder among healthcare staff. Thus, we performed this study to characterize the type of CDG and to advise these ethnic minority parents in Yunnan Province to obtain a prenatal diagnosis in the future.

In conclusion, we have identifiedcompound heterozygous mutations in the genes of *PMM2* (c.241–242 del and c.395 T > C) of a Hani ethnic minority family, which broadens the spectrum of CDGs gene mutations in Chinese patients of Hani ethnic minority.

## Data Availability

The analyzed data sets generated during the study are available from the corresponding authors upon reasonable request.

## Abbreviations

CDGs: Congenital disorder of glycosylation
ID: Intellectual disability
LRT: A likelihood ratio test
PMM2: Phosphomannomutase 2
SIFT: Sorting Intolerant from Tolerant
WES: Whole-exome sequencing
